# Discovery of four novel phenuiviruses from public mosquito RNA-seq data using reference-guided assembly

**DOI:** 10.1128/mra.00447-26

**Published:** 2026-08-11

**Authors:** Thomas F. Leonard

**Affiliations:** 1 Independent Researcher, Alameda, California, USA; Queens College Department of Biology, Queens, New York, USA

**Keywords:** phenuivirus, reference-guided assembly, Phasi Charoen-like viruses, mosquito RNA-seq

## Abstract

We report complete coding sequences of four divergent Phasi Charoen-like viruses from mosquito RNA-seq data sets. Each genome includes L, M, and S segments with minor base ambiguities. These sequences expand known phenuivirus diversity and may aid future research on mosquito vector competence and dengue virus interactions.

## ANNOUNCEMENT

Phasi Charoen-like viruses (PCLVs) are insect-specific members of the family *Phenuiviridae* (genus *Phasivirus*, species *Phasivirus phasiense*), detected in mosquitoes across Southeast Asia (1, 2), China (3, 4), South America (5, 6), India (7–9), and Africa (10, 11). Although they do not infect vertebrates, PCLVs have the potential to modulate mosquito physiology and influence vector competence for arboviruses such as dengue virus (12, 13). Expanding the catalog of PCLV genomic sequences is essential for understanding their evolutionary diversity, ecological distribution, and possible interactions with medically important viruses.

We identified candidate PCLV sequences by aligning publicly available mosquito RNA-seq data sets from the Sequence Read Archive (SRA) against a curated reference panel data set of PCLV sequences generated using makeblastdb (14) (Table 1). Reads were aligned to the reference panel data set using Magic-BLAST (version 1.7.2) (15) with default parameters. SRA data sets showing significant alignment coverage to references were imported into Geneious Prime for reference-guided assembly, alignment visualization, and consensus extraction (16). Assemblies were manually curated to resolve ambiguities and confirm segment structure. We identified four data sets containing coding-complete PCLV, each comprising three segments—long (L), medium (M), and small (S) (Table 1)—with minor coding base ambiguities as follows:

- SRX18712820 (PRJNA911492): 991 adult mosquitoes were collected from eight locations in Yunnan Province, China. Following RNA extraction (TRIzol, Invitrogen) and library generation (MGIEasy mRNA library kit, MGI-Tech), the samples were sequenced with a paired-end (100 bp) protocol using the BGISEQ-500RS sequencing platform (17). Following reference-guided assembly, we recovered all segments with high coverage (Table 1). The L segment contains one ambiguous base at position 5999 (C: 45.4%, T: 54.4%), resulting in a predicted amino acid of threonine or serine in the RNA-dependent RNA polymerase. The M segment contains an ambiguous position at base 1830 (C: 59%, T: 40.8%), leading to possible proline or serine residues at position 598 in the glycoprotein.
- SRX18712865 (PRJNA911492): from the same project (17), we identified an additional data set containing coding-complete L, M, and S segments. The M segment includes one ambiguous base at position 683 (A: 55.4%, G: 44%), resulting in a predicted glutamic acid or glycine residue at position 214 in the glycoprotein.
- SRX25930833 (PRJNA1155297): transcriptomic data set generation on *Aedes aegypti* larvae was performed following RNA extraction (TRIzol), library preparation (Universal RNA-Seq with NuQuant kit, Nugen Tecan Genomics), and sequencing using a paired-end protocol (2 × 50 bp) on a NovaSeq SP flow cell (Illumina) as described in reference 18. This data set yielded coding-complete sequences for all three segments without ambiguous positions.
- SRX26896630 (PRJNA1191412): *Aedes aegypti* colonies used in this study originated from a natural population from Senegal. RNA extraction was performed using TRIzol, and sequencing libraries were prepared with the NEBNext Ultra II RNA library kit (New England Biolabs) as described in reference 19. Libraries were pooled and sequenced on an Illumina NextSeq 550 High Output flow cell with a paired-end 75 base protocol (19). This data set yielded coding-complete sequences for all three segments without ambiguous positions.

**TABLE 1:** Summary of PCLV genome assembly from four SRA data sets, including the reference used for assembly and % similarity to this reference^*a*^

SRA ID	Segment (protein)	Segment length (nucleotides)	GenBank accession	Avg. depth	Max. depth	Reads mapped	Protein length (aa)	Reference accession	Similarity % to reference	GC % of non-gaps
SRX18712820	L (RNA-dependent RNA polymerase)	6,775	BK071769	5,194	10,796	357,566	2,217	MN692603	96.7	38.6
SRX18712820	M (glycoprotein)	3,847	BK071770	6,646	16,723	272,577	1,237	MN692604	96.2	40.1
SRX18712820	S (nucleocapsid)	1,334	BK071771	6,427	17,146	92,840	268	MN692605	96.4	40.1
SRX18712865	L (RNA-dependent RNA polymerase)	6,773	BK074792	9,741.9	22,729	673,373	2,217	MN692603	96.4	38.9
SRX18712865	M (glycoprotein)	3,847	BK074793	16,161.4	29,351	644,306	1,237	MN692604	96.3	39.3
SRX18712865	S (nucleocapsid)	1,334	BK074794	5,424.5	10,126	78,824	268	MN692605	96.8	39.1
SRX25930833	L (RNA-dependent RNA polymerase)	6,773	BK074795	61.3	293	8,250	2,217	MN692603	99.5	38.9
SRX25930833	M (glycoprotein)	3,918	BK074796	102.1	456	7,951	1,237	MN692604	99.6	38.4
SRX25930833	S (nucleocapsid)	1,334	BK074797	83.5	223	2,402	268	MN692605	99.5	37.7
SRX26896630	L (RNA-dependent RNA polymerase)	6,760	BK074798	171.4	325	6,823	2,217	MN692603	98.7	38.2
SRX26896630	M (glycoprotein)	3,836	BK074799	313.3	572	16,512	1,237	MN692604	96.6	38.4
SRX26896630	S (nucleocapsid)	1,337	BK074800	187.3	302	3,689	268	MN692605	97.3	38.6

^*a*^ GC content and GC % of non-gaps found in Geneious assembly statistics panel.

**Fig 1:**
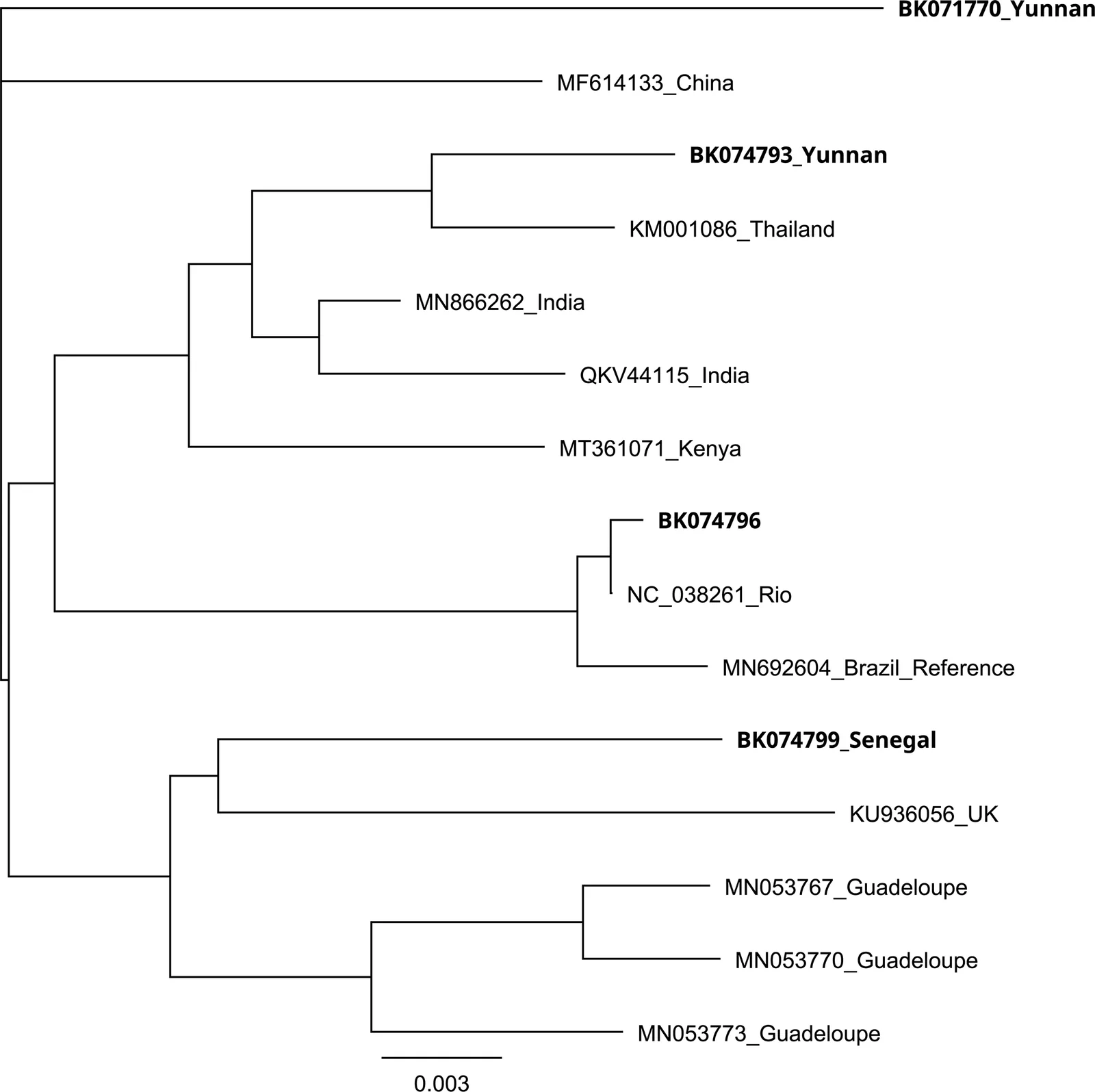
Phylogenetic analysis of the glycoprotein amino acid sequences of four new viral genomes. Tree Construction Methodology: all of the sequences used are from the M segments (glycoprotein). Our four new sequences (bold) and our reference sequence (MN692604_Brazil_Reference) were added to a list of 10 representatives of the Phasi Charoen-like viruses from the work presented in reference 12. These sequences were aligned using Geneious (16), and the tree was generated using the Geneious Tree tool with Alignment Type: global alignment with free end gaps, Cost Matrix: Blosum62, Genetic Distance Model: Jukes-Cantor (21), Tree Build Method: neighbor-joining (22), and Outgroup: no outgroup.

We annotated open reading frames using Geneious Prime’s (16) “Translation” functionality and verified them with BLASTp (20). All sequences show divergence from previously published PCLV genomes, particularly in the M segment, suggesting either geographic variation or previously uncharacterized lineages (Fig. 1). These findings contribute to the catalog of insect-specific viruses and may inform future studies on mosquito viromes and vector-virus interactions.

## Data Availability

The complete coding sequences for all four viruses have been submitted to GenBank and are available under accession numbers in Table 1. Raw RNA-seq data sets are available in the SRA under accession numbers SRX18712820, SRX18712865, SRX25930833, and SRX26896630.
